# RETRACTION: The Investigation and Comparison of the Efficacy and Safety of Stromal Vascular Fraction (SVF), Platelet Rich Plasma (PRP), and 1064‐nm Q‐switched Nd:YAG Laser in Reducing Nanofat Treated Infraorbital Dark Circles and Wrinkles: A Controlled Blinded Randomized Clinical Trial

**DOI:** 10.1111/srt.70289

**Published:** 2025-11-07

**RETRACTION**: M. Roohaninasab, M. Ahmadi, A. Dehghani, S. Zare, A. Goodarzi, M. Nouri, Z. Ebrahimi, E. Behrangi, and M. A. Nilforoushzadeh, “The Investigation and Comparison of the Efficacy and Safety of Stromal Vascular Fraction (SVF), Platelet Rich Plasma (PRP), and 1064‐nm Q‐switched Nd:YAG Laser in Reducing Nanofat Treated Infraorbital Dark Circles and Wrinkles: A Controlled Blinded Randomized Clinical Trial,” *Skin Research and Technology* 30, no. 6 (2024): e13793, https://doi.org/10.1111/srt.13793.

The above article, published online on 20 June 2024 in Wiley Online Library (wileyonlinelibrary.com), has been retracted by agreement between *Skin Research and Technology*; and John Wiley & Sons Ltd. The article was accepted for publication following an insufficient peer review process that does not meet the standards of the journal's peer review policy. Upon further review, it was found that the article contains discrepancies regarding whether appropriate consent was obtained for the publication of identifiable information. Despite multiple attempts to contact the authors for clarification, they have not responded. In light of concerns about the privacy of the individuals involved, the article has been retracted and the corresponding identifiable information has been redacted from the published article. The authors have been informed of the retraction decision but were unavailable for final confirmation.

